# The Surgical Diseases of the Genitourinary Organs

**Published:** 1888-05

**Authors:** 


**The Surgical Diseases of the Genito-Urinary Organs,** including Syphilis. By E. L. Keyes, A. M., M. D., Professor of Genito-Urinary Surgery, Syphilology and Dermatology in Bellevue Hospital Medical College, etc., etc. New York: D. Appleton & Co. 1888.

This is a revision of Van Buren & Keyes's Text Book upon the same subjects, and it will be welcomed very heartily by the profession. The original book was in 1874, the time it was issued, a most admirable and complete treatise on the subject; but since that time genito-urinary surgery has made great advances. Litholapaxy has had its birth, and the supra-pubic operation has been restored to new life and favor. The surgery of the kidney has been constructed anew, and a multitude of minor advances has been made. The distinguished author, from his position as teacher and writer on these subjects, was aided materially in this advance, and we are glad to see that in this new book all obsolete matter has been dropped and so much new matter added that the publishers have found it advisable to set up the entire book anew in type. Among the minor improvements, we note with favor that the author adopts throughout the work the French scale for grading the sizes of instruments. The introduction of an American scale was, we think, due to the writings of Van Buren, but the French scale is now gaining almost universal adoption, and the English and American scales are falling into disuse. The work, as a whole, deserves the highest commendation, and we predict that this edition will be even more popular with the profession than in its time was the original work of "Van Buren & Keyes."

